# A Rare Case of Spontaneous Pneumomediastinum and Pneumopericardium in a 21-Year-Old Male

**DOI:** 10.7759/cureus.83356

**Published:** 2025-05-02

**Authors:** Eleni Giannopoulou, Damianos Tsilivarakis, Stavroula Kosmopoulou, George Lazaros

**Affiliations:** 1 Department of Cardiology, General Hospital of Kalamata, Kalamata, GRC; 2 Department of Cardiology, School of Medicine, Hippokration General Hospital, National and Kapodistrian University of Athens, Athens, GRC

**Keywords:** halo sign, hamman’s sign, paroxysmal cough, pneumomediastinum, pneumopericardium

## Abstract

Spontaneous pneumomediastinum and pneumopericardium, two rare conditions, are mainly described in patients with underlying respiratory illnesses. We present the case of a healthy 21-year-old male who developed these conditions abruptly after a single episode of coughing. Possibly, it was provoked by an increase in intra-alveolar pressure which caused alveolar rupture and air dissemination along the bronchovascular sheath and into the pericardial sac via vulnerable areas. These events led to sustained symptomatology, which prompted the patient to seek medical referral. The presence of air within the mediastinum and pericardium was associated with characteristic auscultatory findings, known as “Hamman’s sign,” and echocardiographic findings, referred to as the “air gap sign.” The condition was diagnosed using a thoracic X-ray, which demonstrated a distinct dark line extending from the level of the left pulmonary artery along the left cardiac contour, curving around its border, a finding consistent with free air in the mediastinum and pericardium. This was subsequently confirmed by thoracic computed tomography. The condition was self-limiting. The patient quickly became asymptomatic, and his vital signs remained normal. No complications or hemodynamic compromise occurred, and he did not require any special medical treatment other than adequate oxygenation. After close monitoring via telemetry and blood examinations, discharge was decided on the third day, following a repeat X-ray, which revealed complete resolution. A follow-up chest radiograph and transthoracic echocardiogram performed two weeks later showed no pathological findings.

## Introduction

The pericardial cavity is enclosed between the two layers of serous pericardium and contains only small amounts of fluid, ranging from 15 to 50 mL, originating from plasma ultrafiltration [1]. Pneumopericardium is characterized by the collection of variable amounts of air or gas within this cavity [2]. It is a rare condition, largely due to the protected position of the heart within the thorax relative to the atmosphere and the limited extent of vulnerable pericardial areas [3]. Air can enter the pericardial space traumatically, typically through injuries to adjacent air-containing structures, such as the respiratory tract or pleural cavity (in cases of pneumothorax), via pericardial rupture [4]. Alternatively, and more commonly, pneumopericardium results from the Macklin effect, wherein traumatic alveolar rupture, after an increase in intra-alveolar pressure, leads to the spread of air along the bronchovascular sheaths into the mediastinum [5]. From there, air may infiltrate the pericardium through vulnerable areas, such as the reflections of the parietal and visceral pericardium near the pulmonary veins [4]. In addition to respiratory tract involvement, cases of pneumopericardium have been reported following communication with the upper gastrointestinal tract, such as iatrogenically, after esophageal stent placement, and as a complication of endomyocardial procedures and pericardiocentesis [2,5-7]. There are also reports of pneumopericardium secondary to traumatic air entry into the subadventitial thoracic or cervical spaces [3]. Due to its common origin, pneumopericardium is often accompanied by pneumomediastinum. However, it may be found in cases of pneumothorax or even pneumoperitoneum, subcutaneous emphysema, or epidural pneumatosis, depending on the route of air dissemination [8].

Less commonly, it can occur atraumatically, following pericardial infections with gas-producing organisms and fistulas connecting the pericardial cavity with air-filled spaces, found in various conditions such as cancer, abscesses, and tuberculosis [4,9]. Recognized predisposing factors for pneumomediastinum, and subsequently pneumopericardium, include smoking and underlying lung disease (chronic obstructive pulmonary disease, asthma, interstitial pulmonary disease) and cases of immense increase in intra-alveolar pressure, such as following cough, vomiting, positive-pressure ventilation, labor, or any Valsalva maneuver and cocaine abuse [4,5,10]. Nevertheless, pneumopericardium may also occur spontaneously after minor elevations of intra-alveolar pressure in otherwise healthy individuals [5]. In such cases, pneumopericardium is rarely isolated and is usually accompanied by air leakage into adjacent compartments such as the mediastinum. This association often signifies greater severity and carries a risk of serious complications, warranting close clinical surveillance [11].

Diagnostically pneumopericardium is a challenging condition with a broad range of symptom severity, depending on the amount of free air in the pericardial sac and the associated underlying pathology. Minor spontaneous cases often go unrecognized due to their subtle symptomatology and clinical findings, complicating the estimation of its true incidence. They are often discovered incidentally on imaging studies [12]. However, severe cases can lead to cardiac tamponade, circulatory collapse, and may present with acute precordial symptoms mimicking myocardial infarction, or produce ST-T changes on electrocardiography that resemble pericarditis [13,14]. In this report, we present a rare case of spontaneous pneumomediastinum and pneumopericardium in an otherwise healthy young adult male, after an episode of paroxysmal cough.

## Case presentation

A 21-year-old male presented to the cardiology emergency department of the General Hospital of Kalamata complaining of constant dull pain at the upper anterior chest wall for 10 hours. The pain reflected to his shoulders bilaterally and to the right lateral cervical area. It started abruptly after an episode of paroxysmal cough due to a feeling of pharyngeal irritation while eating, which forced the patient to bend forward and occurred continuously thereafter. He described the pain as constant in intensity, not significantly influenced by thoracic wall movements, with only mild exacerbation in the supine position and during deep inspiration. The patient denied dyspnea, hemoptysis, or another episode of cough in the near past. His medical and surgical history were unremarkable, and he mentioned no history of lung disease.

Vital signs were within normal limits. On physical examination, there was no palpable tenderness of the thoracic wall. Cardiac auscultation revealed normal heart sounds without murmurs, but a pathological high-pitched crunching sound was audible over the precordium upon firm application of the stethoscope. This sound persisted even during breath-holding, was present both in the supine position and when the patient leaned forward, and resembled “Hamman’s sign,” typically associated with pneumomediastinum and pneumopericardium [9]. The remainder of the physical examination was unremarkable. Standard ECG revealed no pathological findings suggestive of pericarditis or other cardiac disorders. Blood test, however, showed mild leukocytosis (white blood cells: 14,150 /μL, with 81% neutrophils, 13.6% lymphocytes), without other inflammatory markers.

**Figure 1:**
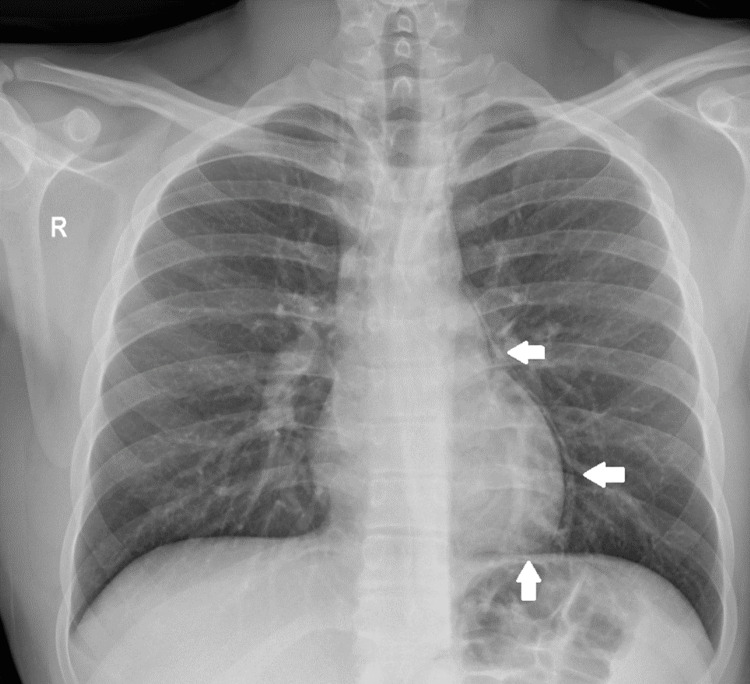
Chest X-ray in the upright position revealing a dark line extending from the level of the pulmonary artery to the apex of the heart and curving around its border (white arrows).

The patient underwent thoracic X-ray, which revealed a thin dark line at the left hemithorax extending from the pulmonary artery down to cardiac apex (Figure 1), suggesting the presence of free air both within the mediastinum and the pericardium. Air was observed above the anatomical limits of the pericardium, at the level of the left pulmonary artery within the mediastinum, and along the inferior cardiac wall directly above the diaphragm, indicating pericardial involvement. No signs of pneumothorax were present. Although our clinical findings were highly suggestive of pneumomediastinum and pneumopericardium, concomitant pneumothorax or pericarditis could not be excluded.

For further investigation a cardiac ultrasound was performed, which showed a normal left and right ventricular ejection fraction, normal chamber dimensions, no significant valvular abnormalities, and normal dimensions of the ascending aorta. A mild pericardial effusion, approximately 0.5 cm, was detected along the inferior cardiac wall. The examination was significantly impaired by artifacts, which were more prominent during ventricular systole, resulting in transient loss of cardiac visualization. These artifacts resolved during diastole on M-mode imaging, a phenomenon known as the “air gap sign,” which supports the presence of pneumopericardium. No signs of cardiac tamponade were observed.

**Figure 2:**
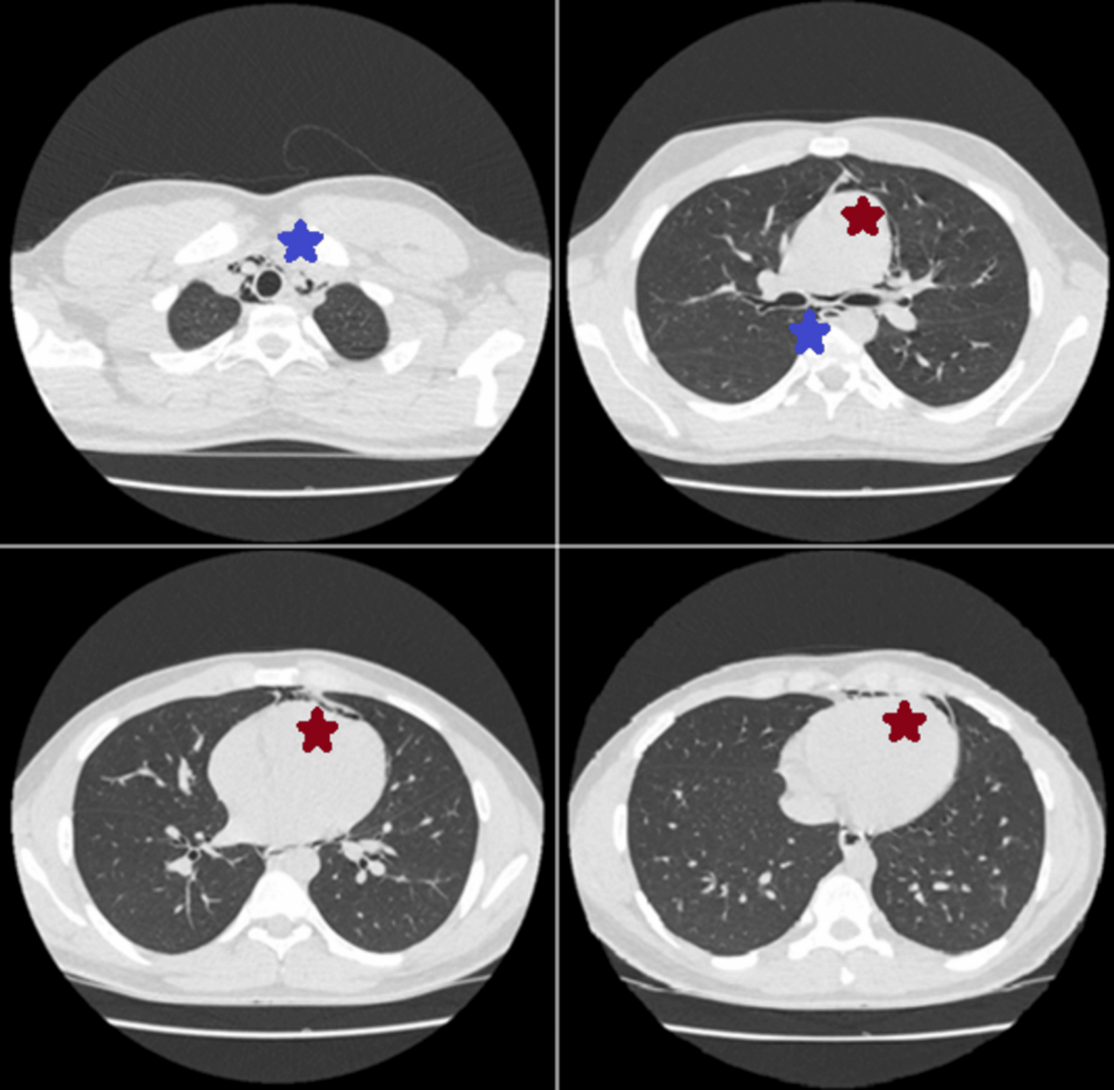
Chest CT showing free air within the mediastinum (blue asterisks) and the pericardial cavity (red asterisks).

Subsequently, we performed a thoracic CT, which confirmed the presence of air in both the pericardial cavity and mediastinum, establishing the diagnosis of spontaneous pneumopericardium and pneumomediastinum, attributed to a brief increase in intra-alveolar pressure during coughing (Figure 2). No other predisposing factors were identified. CT findings indicated that pneumomediastinum was more prominent than pneumopericardium, although both conditions were mild. Given the greater volume of air in the mediastinum, it is possible that air accumulation resulted from the Macklin effect, facilitating the spread of air along the mediastinal fascia and its subsequent entry into the pericardial cavity through vulnerable areas.

Given the clinical stability of the patient, a conservative approach was selected. The patient received low-flow nasal oxygen and analgesics. He was monitored via telemetry and underwent regular blood tests for assessment of myocardial enzymes and inflammatory markers. Throughout his hospitalization, he remained hemodynamically stable and afebrile, with gradual resolution of symptoms. Blood tests remained normal, without elevation of inflammatory markers. On the third day of hospitalization, repeat transthoracic echocardiography demonstrated complete resolution of the pericardial effusion, which was characterized as reactive, given its spontaneous resolution without specific anti-inflammatory treatment and in the absence of signs of inflammation. A new thoracic X-ray showed normalization of the left cardiac border without any other pathologic findings. Because of his clinical improvement, the patient was discharged on the third day. Two weeks later, follow-up chest X-ray and cardiac ultrasound demonstrated no abnormalities.

## Discussion

Pneumopericardium is a rare condition which requires direct communication of the pericardial cavity with adjacent air or gas-containing areas [4]. A communication can be achieved traumatically, iatrogenically, or spontaneously, in non-traumatic origins [9]. Most cases of pneumopericardium are benign in origin, with mild symptomatology or asymptomatic; however, it can be accompanied by breathlessness and precordial pain [15]. Other symptoms include pain that radiates to both shoulders, as reported in our case, back or epigastrium, and palpitations. These symptoms are non-specific for pneumopericardium and largely depend on any underlying pathology, such as the concurrent presence of pneumomediastinum, which mostly presents with symptoms involving the head and neck regions [16]. In our patient, cervical and shoulder pain were likely attributed either to phrenic nerve irritation along its course through the mediastinum due to the presence of air, or to air dissemination along the fascial planes ascending to the cervical region.

On physical examination, percussion of the chest wall may reveal tympani over the area of the heart, and a high-pitched crunching sound that worsens with every heartbeat may be auscultated, the so-called “Hamman’s sign,” which was also detected in our patient [9,15]. Both findings are non-specific for pneumopericardium and can be found in other conditions such as pneumomediastinum [9]. Another auscultatory finding is the “bruit de moulin sign” (mill-wheel murmur), typically described as a splashing sound resulting from the simultaneous presence of air and fluid within the pericardial sac (hydropneumopericardium) [15]. This sign was absent in our case, possibly due to the small volume of both air and fluid collections. In severe cases pneumomediastinum can cause tension and compression of the mediastinal vessels and other anatomical structures, potentially leading to hemodynamic compromise [10]. Similarly, pneumopericardium can also lead to tension and cardiac tamponade [2]. The typical constellation of findings in cardiac tamponade (hypotension, increased jugular venous pressure, blurred heart sounds) caused by fluid effusions differs from that of air origin, as heart sounds are not diminished in cases of air accumulation [17].

Electrocardiographic findings are generally non-specific [15]. In pneumopericardium, echocardiography may demonstrate multiple bands of echoes originating from the anterior cardiac border during cardiac systole, due to higher air concentrations. Between the echo bands cardiac structures remain visible, whereas structures across the bands’ pathway are obscure, the “air gap sign” [18]. This sign, which was observed in our case, resolves during diastole. Another echocardiographic finding described in the literature is the “swirling bubbles sign,” when pneumopericardium is accompanied by pericardial effusion or the absence of an echocardiographic image in large air collections [2].

Diagnosis can be confirmed by chest X-ray in upright position which in cases of pneumopericardium can reveal a dark line of air surrounding the cardiac contour, the “Halo sign” [5]. Unlike pneumomediastinum and pneumothorax, air is only restricted inside the pericardial sac, sparing the thoracic apexes or mediastinum [4]. Its location remains unchanged even after altering patient’s position [9]. According to Triantafyllis et al., the “Halo sign” can also be identified during catheterization, where anteroposterior fluoroscopy reveals a radiopaque space between the cardiac silhouette and the pericardial sac, indicating the presence of pericardial air or fluid [19]. Other findings from chest roentgenogram amplifying the suspicion of pneumopericardium include the “continuous left hemidiaphragm sign” on lateral X-ray and the “continuous diaphragm sign” on frontal view [15]. Ancillary in diagnosis and more appropriate in identifying the etiology is chest CT [17].

Treatment options are individualized and depend on the severity of symptomatology [4]. Due to the benign course of most spontaneous cases, most patients do not require special therapeutic interventions, other than simple observation and administration of low-flow nasal oxygen, as was the case in our patient [17]. Low-flow oxygen therapy is commonly indicated, as it reduces blood nitrogen concentration and increases its diffusion gradient, thereby accelerating the resorption of air from the mediastinum [5]. In cases of bronchospasm or underlying obstructive lung disease, the use of bronchodilators is reasonable to decrease airway resistance and reduce respiratory effort. However, in cases of large collections of pericardial air or hemodynamic instability and manifestations of cardiac tamponade, urgent pericardiocentesis may be mandatory [2,5]. Regarding follow-up, spontaneous pneumomediastinum and pneumopericardium are generally benign conditions with a low risk of long-term recurrence. Asymptomatic patients typically do not require specific outpatient management, unless symptoms reappear following exposure to triggering factors [20]. In our case, the decision to perform imaging follow-up two weeks later was taken as a precaution and may not be deemed necessary in all cases.

## Conclusions

Spontaneous pneumomediastinum and pneumopericardium are rare findings in healthy individuals. In our case, these conditions were triggered by a minor event in a young, healthy male, underscoring the unusual nature of the presentation. It is highly likely that mild cases go unnoticed by healthcare providers, as their clinical presentation may be atypical and may not be clearly identifiable on standard chest X-rays. In such cases, blood tests, ECG, and echocardiography can help exclude potential mimickers such as pericarditis and myocardial infarction. Advanced imaging, particularly CT, can assist in confirming the diagnosis and ruling out other related conditions, such as pneumothorax. Spontaneous pneumomediastinum and pneumopericardium are most often benign and self-limiting, requiring no specific interventions. However, they can present as medical emergencies when they lead to cardiac tamponade or cause significant compression of mediastinal vessels, resulting in hemodynamic instability. Due to the uncertain prognosis, these conditions should always be considered and excluded in patients with thoracic pain, especially following episodes of marked elevation in intrathoracic pressure.
